# Dose variability of supplemental oxygen therapy with open patient interfaces based on in vitro measurements using a physiologically realistic upper airway model

**DOI:** 10.1186/s12931-019-1104-0

**Published:** 2019-07-12

**Authors:** Ira Katz, John Chen, Kelvin Duong, Kaixian Zhu, Marine Pichelin, Georges Caillibotte, Andrew R. Martin

**Affiliations:** 1 Medical R&D, Air Liquide Santé International, Paris Innovation Campus, Les loges-en-Josas, France; 2 Department of Mechanical Engineering, University of Alberta, Edmonton, Canada; 3 Air Liquide Healthcare, Gentilly, France; 4 Technical Innovation, Air Liquide Santé International, Paris Innovation Campus, Les Loges-en-Josas, France

**Keywords:** Oxygen, Nasal cannula, Facemask, Fraction of inspired oxygen, Supplemental oxygen, Oxygen therapy

## Abstract

**Background:**

Supplemental oxygen therapy is widely used in hospitals and in the home for chronic care. However, there are several fundamental problems with the application of this therapy such that patients are often exposed to arterial oxygen concentrations outside of the intended target range. This paper reports volume-averaged tracheal oxygen concentration measurements (FtO2) from in vitro experiments conducted using a physiologically realistic upper airway model. The goal is to provide data to inform a detailed discussion of the delivered oxygen dose.

**Methods:**

A baseline FtO2 dataset using a standard, straight adult nasal cannula was established by varying tidal volume (V_t_), breathing frequency (f), and continuous oxygen flow rate (Q_O2_) between the following levels to create a factorial design: V_t_ = 500, 640, or 800 ml; f = 12, 17, or 22 min^− 1^; Q_O2_ = 2, 4, or 6 l/min. Further experiments were performed to investigate the influence on FtO2 of variation in inspiratory/expiratory ratio, inclusion of an inspiratory or expiratory pause, patient interface selection (e.g. nasal cannula versus a facemask), and rapid breathing patterns in comparison with the baseline measurements.

**Results:**

Oxygen concentration measured at the trachea varied by as much as 60% (i.e. from 30.2 to 48.0% of absolute oxygen concentration) for the same oxygen supply flow rate due to variation in simulated breathing pattern. Among the baseline cases, the chief reasons for variation were 1) the influence of variation in tidal volume leading to variable FiO2 and 2) variation in breathing frequency affecting volume of supplemental oxygen delivered through the breath.

**Conclusion:**

For oxygen administration using open patient interfaces there was variability in the concentration and quantity of oxygen delivered to the trachea over the large range of scenarios studied. Of primary importance in evaluating the oxygen dose is knowledge of the breathing parameters that determine the average inhalation flow rate relative to the oxygen flow rate. Otherwise, the oxygen dose cannot be determined.

**Electronic supplementary material:**

The online version of this article (10.1186/s12931-019-1104-0) contains supplementary material, which is available to authorized users.

## Introduction

Supplemental oxygen therapy is widely used in hospitals for acute care and in the home for chronic care. Oxygen is one of the most commonly administered medications in patients receiving emergency or hospital-based care [1]. In the United States alone, more than 1 million patients receive home oxygenation therapy [2].

Despite this widespread use, potential hazards due to inaccurate dosing causing hypoxia and hyperoxia have been identified [3]. For example, it has been shown that toxic reactive oxygen species in lung capillary endothelial cells increased with hyperoxic ventialation of 70% oxygen [4]. On the other hand, many babies died due to inadequate oxygenation to avoid retrolental fibroplasia [5]. Over that last 10 years or so there have been several surveys of clinicians in various countries and disciplines to document their comprehension of oxygen therapies [6, 7]. Furthermore, other clinical studies have reported on the actual practices employed and the success of these practices in maintaining adequate oxygenation as measured by blood gas analysis or pulse oximetry (resulting in a peripheral capillary oxygen saturation, SpO2, measurement) [8–21]. The essence of these studies can be gleaned from the conclusion written by Helmerhorst and colleauges, that most ICU [Intensive Care Unit] clinicians understand the danger of prolonged exposure to hyperoxia; however, they report higher arterial oxygen levels than target ranges [6]. Furthermore, for those patients receiving supplemental oxygen in the home there are frequent and varied problems, particularly a lack of access to effective instruction and adequate portable systems. Thus, there is a need to promote access to equipment and services tailored to each patient’s needs [2].

Consider the conviction, which is also the regulatory norm, that *oxygen is a drug* [1, 22]; as correct dosing is a fundamental aspect in all drug administration, it is a topic of inherent interest for supplemental oxygen therapy. As a step in the direction of a better overall understanding of the dose associated with the administration of supplemental oxygen therapy, in this paper tracheal oxygen concentration measurements from in vitro experiments using a physiologically realistic upper airway model are reported. The in vitro methodology follows that recently presented by Chen et al. [23, 24] to compare pulsed oxygen delivery from portable oxygen concentrators to continuous flow oxygen delivery. More generally, in vitro methods incorporating realistic or idealized upper airway geometries have been widely used in characterizing inhalation drug delivery systems [25–28].

Here our results are presented in terms of average oxygen concentration percentage at the trachea (FtO2) compared to the average oxygen concentration inhaled (FiO2), as well as the volume of oxygen delivered per breath and per minute.

## Methods

### Conceptual framework

The conceptual framework for this study is to address the passage of supplemental oxygen mixed with air at the patient interface and through the upper airway to the trachea. In this paper, the key locations where the dose is assessed are at the interface (an estimate) and at the trachea (a measurement). In previous papers, under different conditions the passage through the tracheobronchial tree to the terminal bronchiole and the transfer to blood (oxygen flux, VO2) have been considered, but these are beyond the scope of the present paper, which is to highlight the variability in oxygen dosing occurring before supplementary oxygen reaches the gas exchange regions of the respiratory system [23, 29, 30]. However, these tracheal measurements can be considered as boundary conditions for further analysis of the complete transport to the blood that would accurately combine and characterize both gas transport in the upper airways and gas exchange.

### Interface estimate

For all of the cases considered herein (with the exception of the Oxyarm interface, discussed below) the oxygen dose being delivered at the patient interface to the airway opening is estimated by taking the flow-weighted average concentration between the ambient inhaled air and the supplemental oxygen flows. Eq. 1 is based on the assumptions that the total average inspiratory flow rate is tidal volume *V*_*t*_ divided by inspiratory time *t*_*i*_, and that the ambient air infiltration (*Q*_*air*_) is the total average inspiratory flow less the supplemental oxygen flow (*Q*_*sup*_). Eq. 2 is the estimated FiO2 in the total (mixed) inspiratory flow entering the model airway:

1 $$ {Q}_{air}=\frac{V_t}{t_i}-{Q}_{sup} $$

2 $$ FiO2=\frac{21{Q}_{air}+100{Q}_{sup}}{Q_{air}+{Q}_{sup}} $$

The estimated volume of inhaled oxygen is then*, ViO*2 = *FiO*2 ∗ *V*_*t*_*./100*

### In vitro model

**Fig. 1:**
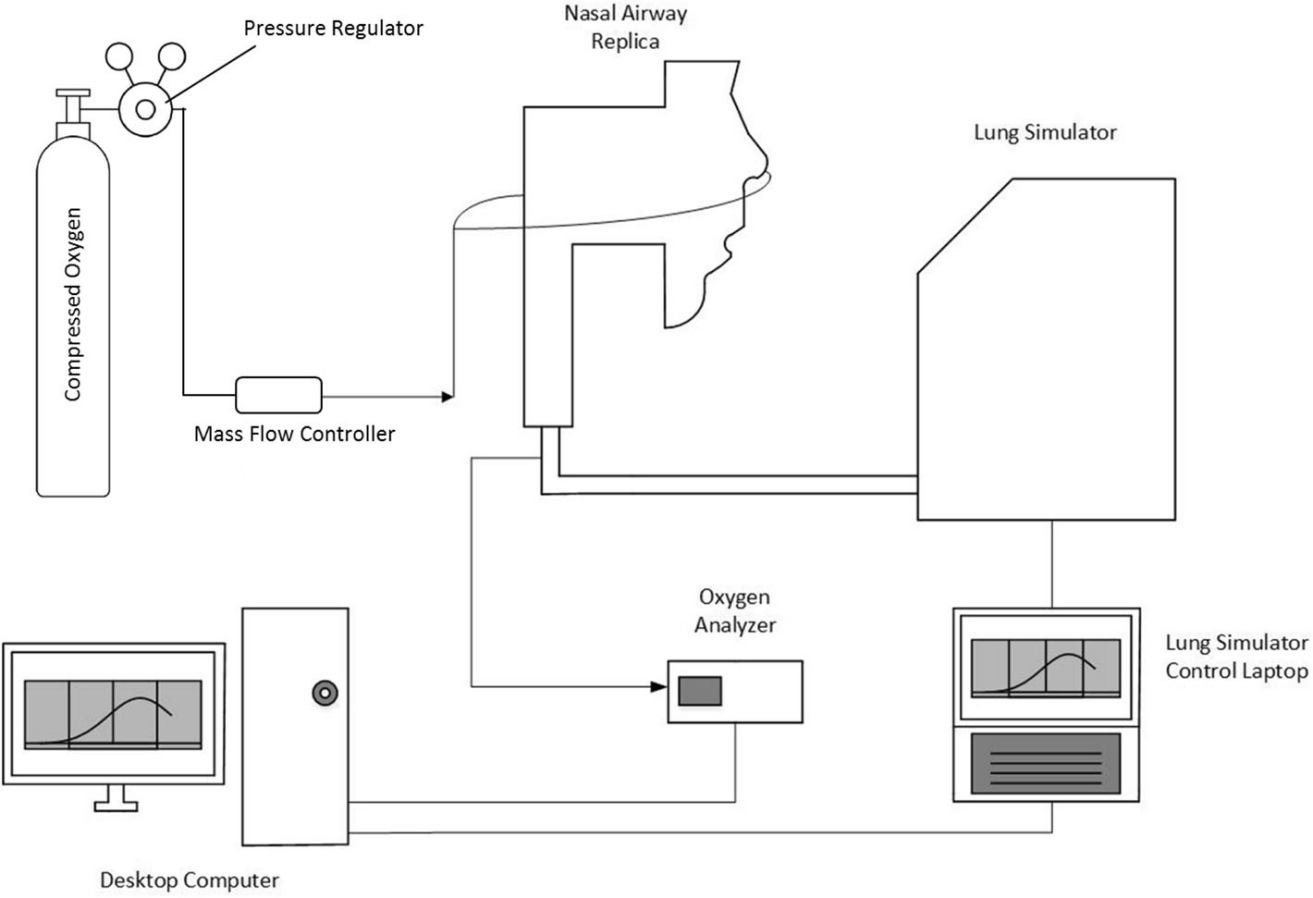
Schematic and photo of the experimental apparatus. Arrows indicate direction of gas flow on the schematic [23]

The general in vitro methodology is described by Chen et al. [23, 24]. Briefly, with reference to Fig. 1, a realistic adult nose-throat airway replica was connected to a lung simulator (ASL 5000 Breathing Simulator, IngMar Medical USA) used to simulate tidal breathing. A photo of the experimental setup is also shown in Fig. 1. The volume of the chamber of the test lung was recorded during simulated breathing at a sampling frequency of 512 Hz using the ASL 5000 software. The airway replica was developed previously using medical images from an adult volunteer and 3D-printing techniques [31]. The replica outlet, corresponding to the entrance to the trachea, was connected to the lung simulator breathing circuit with 22 mm internal diameter tubing. The total internal volume of this tubing was 135 cm^3^, representative of the conducting airway volume from the trachea to the gas exchange regions of the lungs for an average adult with 3 L functional residual capacity. Continuous flows of oxygen were delivered to the replica via nasal cannula (or other patient interfaces as indicated) from a regulated compressed oxygen cylinder through a calibrated mass flow controller (MCMC-Series Mass Flow Controller, Alicat, USA). Gas was sampled at the exit of the replica (representing the trachea) and oxygen concentrations were measured using a laser diode analyzer (GA-200, iWorx, USA) at a frequency of 34.88 Hz.

Real-time oxygen concentrations from the analyzer and volume vs. time data from the lung simulator were streamed via virtual instrument software architecture (VISA) serial and TCP/IP connection ports, respectively, to a program written in LabVIEW (National Instruments, USA) for collection and synchronization. Total gas flows at each point in time were then calculated using forward differences from the volume data. To synchronize the two signals (oxygen concentration and flow), we initially allowed a continuous stream of oxygen to bleed into the apparatus under a condition of zero flow. Once we were able to observe an increase in the oxygen concentration over time at the trachea, we induced an inspiratory effort using the test lung, resulting in a sudden jump in the oxygen concentration. The two signals could then be synchronized by matching the time of the start of the inspiratory flow with the jump in oxygen concentration. The synchronized flow and oxygen concentration waveforms were consistent with oxygen waveform features (e.g. presence of the anatomic reservoir at the start of inspiration) previously described in the literature [32]. In addition, this synchronization method was able to minimize the difference between the total inhaled and exhaled volumes of oxygen. During steady state with ideal synchronization, this difference would be zero, given that the in vitro experimental setup did not include an oxygen uptake mechanism.

The flow rate of oxygen passing through the trachea over time was calculated by multiplying inspiration flow with measured oxygen concentrations at the same time point. Inspiration start and end times were identified as times of zero flow rate. These oxygen flow rates were then numerically integrated using the trapezoidal rule from the start to the end of inspiration to determine a volume of oxygen inhaled per breath. Tidal volume, V_t_, was calculated by integrating inspiration flow rates over the inspiratory time period. The volume of inhaled oxygen was then divided by V_t_ to obtain a volume-averaged value for that breath, which we call here FtO2 to represent the concentration of gas passing the trachea during inhalation. FtO2 for each test was calculated as the average of five consecutive breaths after a steady state in expiratory oxygen concentration was observed (after at least 50 breaths).

**Fig. 2:**
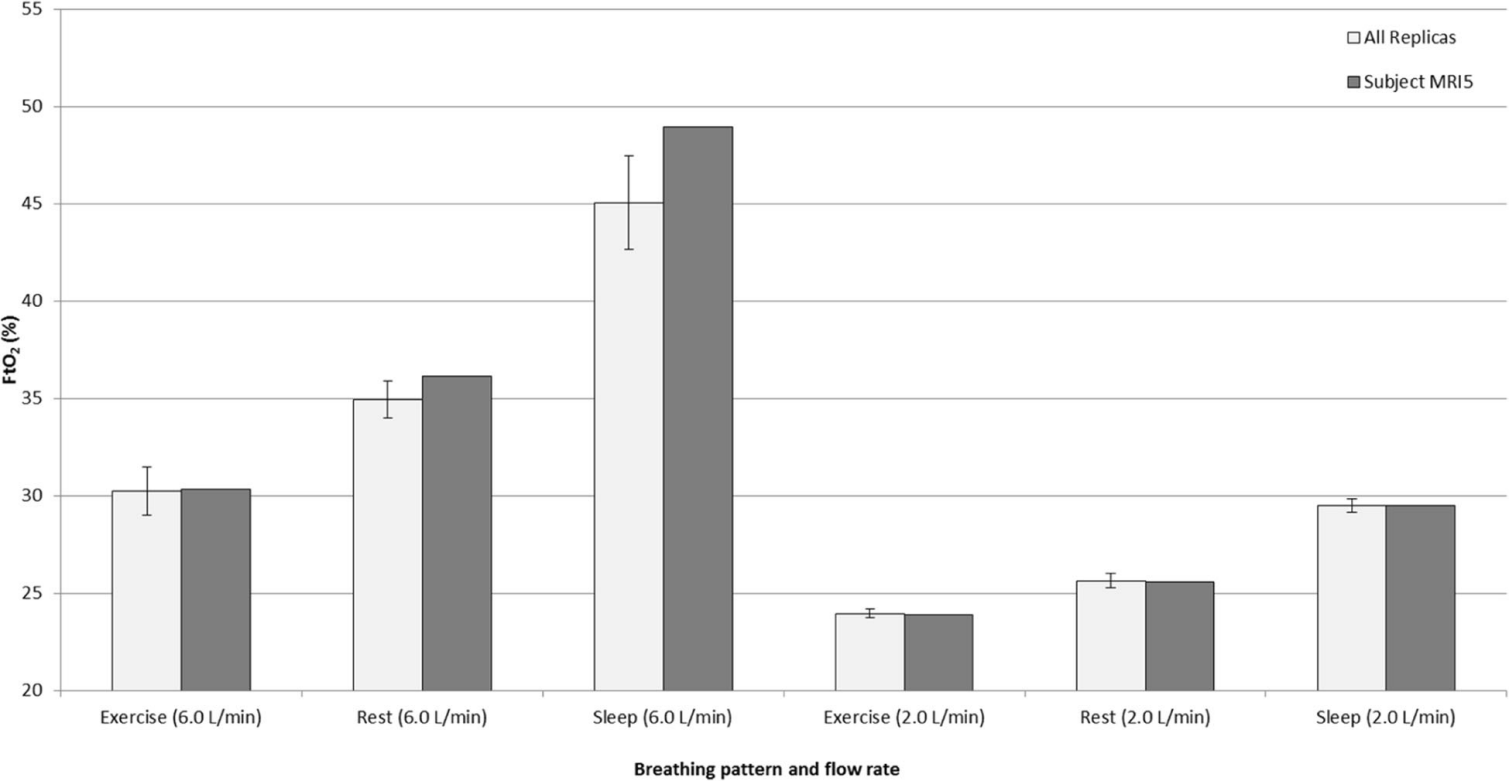
Comparison of FtO2 measured in nose-throat replica MRI5 vs. average data obtained from the larger set of 15 adult nose-throat replicas. Error bars denote one standard deviation. Exercise, Rest, and Sleep breathing patterns are described in Chen et al. [23]. Data labels ‘2.0 L/min’ and ‘6.0 L/min’ refer to tests performed with continuous oxygen delivery at the flow rate indicated

Previous testing of both continuous and pulsed oxygen delivery through realistic adult nose-throat airway replicas has been reported by Chen et al. [23] In that work, tests were repeated in 15 replicas, and FtO2 data were presented as average values along with standard deviations between replicas. These tests provide a subset of data through which the influence of nasal airway geometry on FtO2 may be explored. For further testing described herein, a single replica has been selected, to allow a wider range of breathing parameters to be explored while limiting the total number of experimental repetitions. Fig. 2 displays FtO2 values measured for the replica selected for the present study (labeled MRI5) compared with average values for the 15 replicas studied by Chen et al. [23]. It can be seen that FtO2 measured in the selected replica was in reasonable agreement with the average for the flow rates and breathing patterns previously evaluated. Also of note is the relatively small magnitude of error bars reflecting standard deviation between airway geometries previously studied. This suggests that the large intersubject variability in inhaled oxygen concentrations reported by previous authors [33, 34] may not be due to variability in upper airway geometry, but rather due to uncontrolled variability in breathing pattern, and supports the use of a single upper airway geometry in the present experiments.

The design of the experiments followed a philosophy to mimic physiology as much as possible within the limits of a mechanical system. The strength of in vitro bench model is that it is highly repeatable and allows for the parametric controlled experiments that are reported herein. Thus, the error analysis we employed follows a different approach than would be the case for in vivo human testing, where variability between subjects is comparatively large. That is, in lieu of a statistical analysis, repeatability assessments were conducted for all baseline parameter combinations for the baseline tests described below across five simulated breaths and for experimental repetitions conducted over three separate days, which included repositioning of the cannula between repetitions. The average standard deviation of FtO2 among all the tests was 0.3% oxygen reading. Accordingly, the controlled bench experiments described herein were highly repeatable, and experimental uncertainty was deemed negligible relative to the variation in FtO2 between experiments.

### In vitro dosing experiments

The apparatus and techniques were applied to 105 separate in vitro dosing experiments consisting of a baseline set plus several other sets to investigate fundamental oxygen therapy variables. Thus, the purpose of the baseline dataset is to provide a basis of comparison for the other oxygen therapy variables considered in a parametric methodology; such that we report the oxygen dose first for the baseline cases and then as more or less from the baseline for the single variable that was modified. Additional file 1: Table S1 provides a list of each experiment’s parameters. A description of the experiment sets follows.

### Baseline dataset

The baseline dataset was established using a standard, straight adult nasal cannula (Model 1103; Hudson RCI, USA) patient interface and by varying tidal volume (V_t_), breathing frequency (f), and oxygen flow rate (Q_O2_) between the levels specified below in a factorial design: V_t_ = 500, 640, or 800 ml; f = 12, 17, or 22 min^− 1^; Q_O2_ = 2, 4, or 6 l/min. The inspiratory/expiratory (i/e) ratio was held constant at approximately 0.5 to match inhaled and exhaled volumes in user-defined breaths simulated by the ASL 5000 instrument (i/e deviated slightly from 0.5 as indicated in Additional file 1: Table S1 with no end-expiratory pause nor end-inspiratory pause in the simulated breaths). Inspiratory and expiratory phases of the breath are modeled as half-sine waves.

### Influence of rapid breathing

For patients that develop respiratory failure, Sim et al. [35] report that there is no universal breathing pattern, and that there is little in the scientific literature regarding breathing patterns during respiratory failure. However, they relate clinical observations that a pattern of high respiratory rate with small tidal volumes is often seen in respiratory distress syndrome, left ventricular failure, diaphragmatic splinting, and pulmonary fibrosis. Separately, Bateman and Leach [36] provide a representative breathing pattern for a patient undergoing respiratory failure with V_t_ = 750 ml and f = 40 min^− 1^, indicating rapid but not shallow breathing. In many cases, patients experiencing respiratory failure may receive high flow oxygen through nasal cannula or face mask, where FiO_2_ may be less influenced by breathing pattern. However, as some patients may receive low-flow oxygen delivery through a nasal cannula, tests with simulated rapid breathing representative of the above clinical observations were performed. Testing was conducted with V_t_ of 250 ml and 750 ml, f = 30 min^− 1^, i/e = 1.0, and Q_O2_ of 2, 4, and 6 l/min.

### Influence of deviations from characteristic breathing pattern

Three deviations from the characteristic baseline breathing pattern were performed i) for an inspiratory/expiratory ratio i/e = 1.0, ii) an i/e ratio of 0.5, and with the addition of an end-expiratory pause (EEP) occupying 20% of the total breath time, and iii) with an i/e ratio of 0.5, and with the addition of an end-inspiratory pause (EIP) occupying 10% of the total breath time.

For each type of deviation from the characteristic breathing pattern, a subset of tests were repeated from the baseline dataset. The repeated combinations of tidal volume and breathing pattern were V_t_ = 500 ml and f = 12 min^− 1^, V_t_ = 640 ml and f = 17 min^− 1^, and V_t_ = 800 ml and f = 22 min^− 1^. Each of these combinations was evaluated with Q_O2_ of 2, 4, and 6 l/min, for the three deviations from the characteristic breathing pattern outlined above.

### Influence of patient Interface

Five additional patient interface types besides the standard cannula used in the baseline tests were evaluated i) a flared cannula (1104; Hudson RCI, USA), ii) a second model of straight cannula (1600Q-7-50; Salter Labs, USA), iii) a simple oxygen mask (GK 1041; Glenwood Labs, Canada), iv) the Oxymask (OM-1125-8; Southmedic, Canada) where testing was performed with the cuff sealed to the replica face using adhesive putty, and v) the Oxyarm (OA-PLUS-1125-8; Southmedic, Canada) where the oxygen outlet was positioned above the replica’s face without contact following the manufacturer’s illustrative instructions. For each patient interface, experiments were performed for three combinations of tidal volume and breathing pattern: V_t_ = 500 ml and f = 12 min^− 1^, V_t_ = 640 ml and f = 17 min^− 1^, and V_t_ = 800 ml and f = 22 min^− 1^. Each of these combinations was evaluated with Q_O2_ of 2, 4, and 6 l/min.

## Results

### Baseline experiments

The results in terms of oxygen concentration for the 27 baseline experiments that cover a range of breathing patterns and oxygen supply flow rates are given in Additional file 1: Table S1. The data presented in terms of oxygen concentration, oxygen flux VO2 and volume of oxygen delivered per breath estimated at the interface and measured at the trachea. Data for all 105 cases are also provided in the same table.

**Fig. 3:**
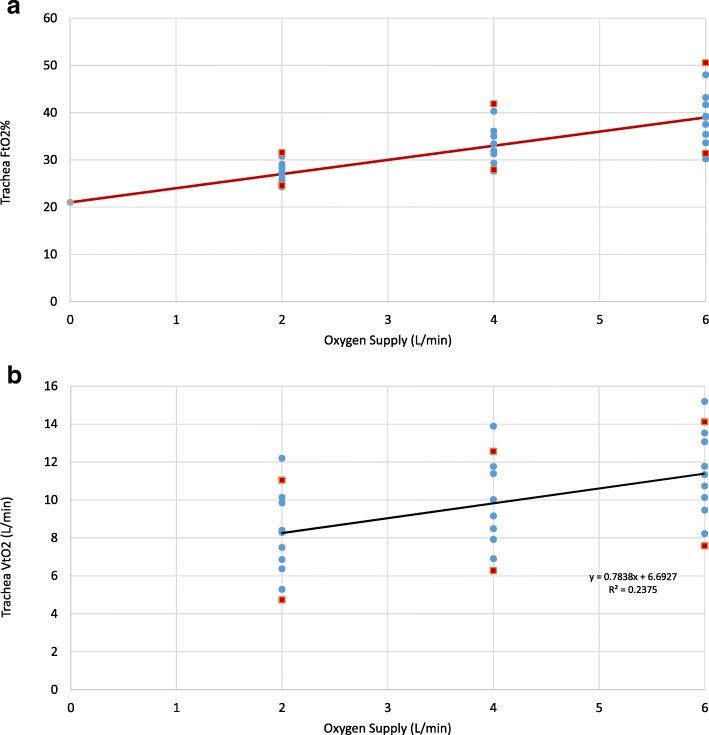
**a** Tracheal oxygen concentration (FtO2) as a function of the supplemental oxygen supply flow rate for the baseline experiments (circles) and for the rapid breathing cases (squares). The solid red line represents a typical clinical estimate of FiO2 as is 21 + (L/min O_2_ * 3)) [37, 38]. Note that the regression line is not visible behind the clinical estimate. **b** Trachea oxygen flow (VtO2) as a function of supplemental oxygen supply flow rate for the baseline experiments, circles, and for the rapid breathing cases, squares

**Fig. 4:**
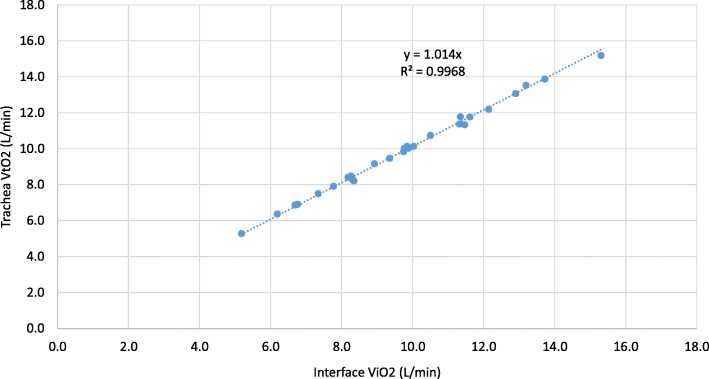
Trachea VtO2 as a function of the linear estimate of the interface ViO2 for the baseline experiments

**Fig. 5:**
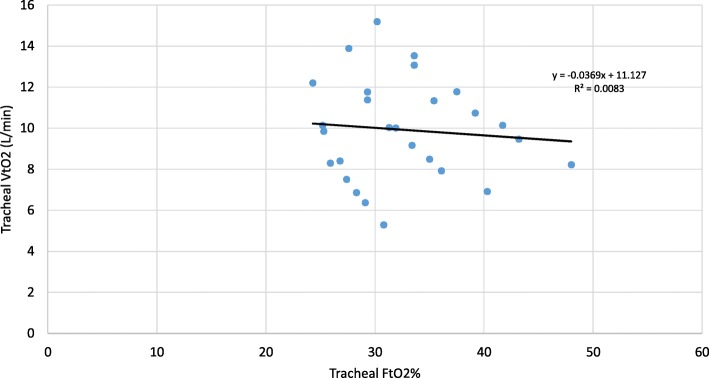
Trachea VtO2 as a function of the trachea oxygen concentration for the baseline experiments

**Fig. 6:**
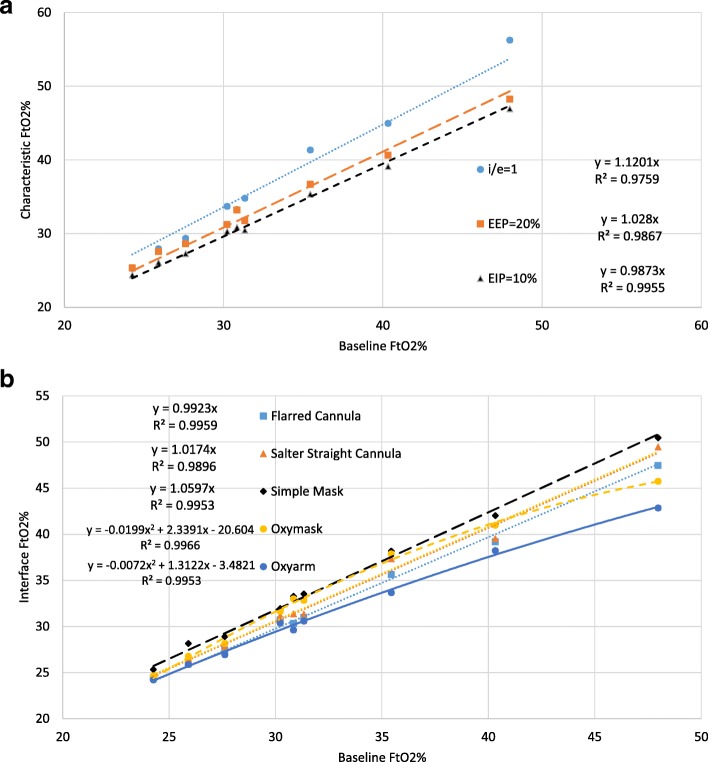
**a** The effect of characteristic breathing pattern deviation on tracheal oxygen concentration for the baseline subset experiments. **b** The effect of patient interface on tracheal oxygen concentration for the baseline subset experiments

The dose variability can be interpreted through the plots shown in Figs. 3, 4, 5 and 6. As shown in Fig. 3a, there is substantial variation (a relative variation by as much as 60%, from 30.2 to 48.0% of oxygen, at the same 6 L/min oxygen supply flow rate) in oxygen concentration measured at the trachea for the same oxygen supply flow rate. This result underscores the serious drawback to prescribing supplemental oxygen therapy based solely on the oxygen supply flow rate. A typical estimate of FiO2 [37, 38] as indicated by the solid red line is 21 + (L/min O_2_ * 3). By sight, it follows the average trend accurately, but cannot account for the variations (for the regression line that overlaps it R^2^ = 0.49). Fig. 3b indicates that this drawback of variability in dose based on oxygen supply flow rate persists if the dose is considered as volume/time; i.e., in L/min. In fact, the relative variability in volume/time reaches 130% at the 2 L/min supply flow rate.

Figure 4 shows experimentally measured VtO2 as a function of the estimated oxygen minute ventilation ViO2. The slope of the trend line, 1.0132, shows that the estimate is a very good indicator, but also that the trachea measurement is marginally greater than the estimate. One source of increased oxygen at the trachea is due to pooling (a breathing frequency effect) in the upper airways that is not accounted for in the estimate [32]. It is important to recognize, that while the estimate itself is accurate, it requires knowledge of the inhalation flow rate, derived from the tidal volume and inspiratory time (see Eqs. 1 and 2), parameters that are not generally known for patients using open interfaces. This result is in consonance with the recent paper by Dupriez et al. that showed the importance of inhalation flow rate on delivered oxygen fraction [39].

In Fig. 5 the tracheal VtO2 is compared to the tracheal concentration FtO2%. It is noteworthy that for the same oxygen concentration the amount of oxygen actually passing the trachea can be very different because of variation in minute ventilation. For example, at about 31% concentration the VtO2 varies between 2 L/min and over 5 L/min.

### Rapid breathing

The rapid breathing cases are indicated by squares in Fig. 3. The low tidal volume cases resulted in high FtO2% but low VtO2 in comparison to the baseline cases. To the contrary, the high tidal volume cases resulted in low FtO2% but high VtO2.

### Influence of characteristic breathing pattern deviation

To investigate the effect of deviations from the characteristic breathing pattern used for the baseline set of cases, for a subset of cases the inspiratory/expiratory (i/e) ratio was changed from 0.5 to 1. Additional subsets of cases were studied with i/e maintained at 0.5 but with the addition of an end expiratory pause (EEP) of 20%, or an end inspiratory pause (EIP) of 10%. These data, evaluated in terms of the measured FtO2%, are plotted versus the baseline case with equivalent tidal volume and frequency in Fig. 6a. From the slopes of the trend lines it can be observed that increasing i/e ratio increases oxygen concentration (1.12 > 1), in contrast adding the EEP or EIP had much less effect (1.028~1; and 0.987~1). Changing the i:e ratio has a more significant influence on FtO2 than adding EEP because variation in the inspiratory time has a greater influence of FtO2 than does the pooling effect.

### Influence of patient Interface

FtO2 measured for the subset of baseline cases with five additional patient interfaces is compared to the baseline cases using the Hudson straight cannula in Fig. 6b. For the two additional nasal cannula types and the simple mask, a linear trend line fits the data relatively well. For these cases it can be seen that there is very little difference in FtO2 between the Hudson model straight cannula used for the baseline experiments and the Salter model straight cannula (slope of 1.0174). For the flared cannula as well there was very little difference in results for FtO2% (slope of 0.9953). This suggests that commentary presented herein can be applied to low flow oxygen delivery via nasal cannula in general, and are not specific to any particular make or model of nasal cannula. Using a simple mask, the FtO2 was higher than for nasal cannula by about 6% relative (slope of 1.0597). For the Oxymask and Oxyarm a quadratic trend line fit the data better than a linear fit. Both of these unique interfaces tend to provide similar FtO2 as do nasal cannula at lower flow rates, but tend to underperform as the flow rate, and hence FtO2, increases.

## Discussion

Results of volume-averaged tracheal oxygen concentration measurements (FtO2) from in vitro experiments conducted using a physiologically realistic upper airway model are reported. These data indicate that using a standard cannula and over a wide range of breathing parameters and oxygen flow rates, there is significant variability is the oxygen dose delivered to the trachea. This is true in terms of the concentration volume percentage and in the volume of oxygen delivered per breath. These data formed a baseline for the comparison of further changes in breathing (such as inspiratory to expiratory ratio) and patient interface (e.g., a simple mask). It is also shown that if the respiratory parameters were known, in particular the inspiratory flow rate, the oxygen dose could be accurately characterized. This is consistent with recent in vitro results presented by Duprez et al. [39]; however, the present experiments advance in vitro methodology through incorporation of a realistic upper airway geometry that allowed several patient interfaces to be investigated. As shown by Fig. 4, the linearized or average inspiratory flow rate, based on tidal volume and inspiratory time will suffice to make accurate predictions, though the instantaneous values integrated over the inspiratory time would be more rigorously accurate. Consistent with the conclusions of Duprez et al. [39], our results indicate that prediction formulas commonly used by clinicians, which do not include inspiratory flow rate, could lead to over- or under-prediction of inspired oxygen fractions (Fig. 3a).

The context of the results presented herein can be considered in regard to a recent review paper: Walsh and Smallwood [40] provided a captivating historical synopsis of oxygen treatment. Although their focus was on pediatric treatment, several historical milestones had similar influence on the treatment of adults. For many decades oxygen administration dosing was based on the observation of skin color, as well as the breathing frequency, regularity, and perceived work of breathing. It was not until the 1960s that sampling of blood gases became common practice in hospitals. More recently, pulse oximetry has become widely available to provide a simple to obtain measure of oxygenation, though with some limitations in accuracy that can be consequential [40, 41]. For example, due to artifacts arising from low perfusion, noise, or motion, and because of the sigmoid shape of the oxyhemoglobin dissociation curve, pulse oximetry may not detect hypoxemia in some patients [41, 42].

Walsh and Smallwood note that the overall goal of oxygen therapy is to achieve *adequate oxygenation* using the lowest fraction of delivered oxygen. However, achieving this goal is complicated by several factors. Foremost is that despite more than 75 years of routine oxygen administration, normoxia (administration that avoids the detrimental effects of hypoxia on the one hand and those caused by hyperoxia on the other) is not clearly or universally defined [43–45], leading to wide variations in practice [46]. Even the definition of *adequate oxygenation* is not clear [47]. Certainly adequate oxygenation implies a balance between oxygen delivery to the tissues and their rate of oxygen consumption. However, another definition may include oxygen delivery that allows the cells to consume oxygen for energy normally; otherwise anaerobic metabolism or cell death can occur, depending on cell type [40].

Another key to maintaining adequate oxygenation is the understanding that there are two components that contribute to oxygen uptake from the alveoli: oxygen carrying capacity (e.g., hematocrit) and perfusion (blood flow). Thus, a patient can have adequate oxygen carrying capacity but low cardiac output and suffer from inadequate oxygen uptake, as well as the problems associated with inadequate ventilation in the lungs. Beyond patient variability due to underlying pathology, other physical characteristics (e.g., sex, age, size) may also influence oxygen dose. Furthermore, variability in the application of oxygen therapy by clinicians suggests that they in some cases lack adequate knowledge in the use of oxygen delivery equipment [48]; as well as in the concepts of oxygen delivery and equipment used to monitor the effects of oxygen therapy [49]. Walsh and Smallwood maintain that “unfortunately, adverse reactions from the therapeutic use of oxygen are not well documented in pediatric patients. Therefore, it is imperative that oxygen therapy be provided at accurate and safe levels with the lowest possible fractional concentration of inspired oxygen (FiO2)” [40]. These concerns also apply to adult patients. Indeed, in a recent review article Branson [50] noted that “The role of oxygen in COPD exacerbation has the ability to be therapeutic and toxic”.

The recognition of these issues had promoted initiatives to improve supplemental oxygen therapy. One such initiative that reflects the state-of-the-art is a 2017 guideline promulgated by the British Thoracic Society [51]. The key aspects of the guideline related to this paper are: 1) “The essence of this guideline can be summarised simply as a requirement for oxygen to be prescribed according to a target saturation range and for those who administer oxygen therapy to monitor the patient and keep within the target saturation range”; and 2) “The oxygen saturation should be checked by pulse oximetry in all breathless and acutely ill patients, ‘the fifth vital sign’ (supplemented by blood gases when necessary), and the inspired oxygen concentration should be recorded on the observation chart with the oximetry result. (The other vital signs are pulse rate, blood pressure, temperature and respiratory rate)” [51].

In parallel to the new recognition that supplemental oxygen therapy should be guided by a SpO2 target has been the development of devices to control oxygen delivery for closed inhalation circuits based on algorithms relating SpO2 to the inhaled oxygen fraction, FiO2 [49, 50]. In effect such devices provide the feedback automatically that otherwise is performed by clinicians. For an open inhalation circuit (e.g., nasal cannula, facemask) the FiO2 can be estimated based upon an initially selected flow rate of 100% oxygen based on well understood methods available in respiratory therapy textbooks (e.g. [52]). However, as demonstrated above (Fig. 3), variability around such estimates is considerable due to variations in breathing pattern. Thus, the inspired oxygen concentration cannot be recorded such that clinicians in practice are left to beg the question: what oxygen flow rate will maintain a particular FiO2 that in turn will provide a target SpO2? There now exist automatic feedback control devices for open patient interfaces that control the supplemental oxygen flow rate in response to the SpO2 target [3, 53]. However, as shown above, controlling only the supplemental oxygen flow rate does not control the actual delivered dose of oxygen.

These difficulties with the understanding of dose are rooted in the complex physiology and pathology related to insufficient oxygenation. In West’s [54] canonical text on respiratory physiology perfusion limited and diffusion limited uptake to the blood are discussed. These limitations are related to the number of oxygen molecules that can pass from the gas phase into the blood. Furthermore, the effect of ventilation-perfusion inequality on overall gas exchange is reviewed in a separate chapter. This ventilation limitation is in effect a lack of oxygen molecules present in some alveoli. Supplemental oxygen therapy has very little effect on perfusion nor diffusion limitations, but is clearly aimed to alleviate a deficiency in the presence of oxygen molecules in the alveoli. Thus, it is possible that better knowledge of the dose could lead to better understanding of the manifestation of pathology for individual patients in their response to supplemental oxygen therapy.

The definition of a dose is the quantity of drug to be taken within a given time period. It is evident that specifying the oxygen dose using SpO2 target range falls outside this definition, because neither a specific quantity nor time period are indicated. Thus, a more relevant specification is VO2. Indeed, from a physiological perspective, VO2 is the most relevant variable. For example, Ye et al. [55] note that oxygen uptake kinetics (changes in VO2) “is an important physiological parameter for the determination of functional health status and muscle energetics during physical exercise” [56]. In addition, the VO2 provides a useful assessment of the body’s ability to support a change in metabolic demand and an insight into the circulatory and metabolic response to exercise. Several studies confirmed that oxygen consumption is mainly controlled by intramuscular factor related metabolic system [57, 58]. Different from heart rate, the oxygen uptake cannot be affected by mood, stress, etc., and is generally considered as the most accurate measurement of the fitness for the cardiorespiratory system [59, 60].

It can be supposed, that a better understanding and determination of the true dose all along the route of administration as discussed herein should provide better tracking toward the SpO2 target. In the sense of comparing the oxygen content in the blood to the oxygen delivered, the ratio of SpO2/FiO2 is providing guidance for more effective oxygen delivery [61]. However, the problem of accurately estimating FiO2 when using open interfaces has been identified [62, 63]. Another promising direction for research would recognize the importance of VO2, such that the deviation of SpO2 could be tracked to where the VO2 was deficient in order to pinpoint manifestations of pathology.

The present study may have a broader application, in that it could be extrapolated to better understand the administration of other gases such as nitrous oxide and nitric oxide. That is, the fundamental respiratory physiology will also have a profound effect on the open circuit administration dose of these other gases.

There are several limitations to this study. While a large number of scenarios have been considered, the study is not exhaustive. Notably not all types of patient interface were tested, such as venturi and rebreather masks. The in vitro model only considers inhalation with a closed mouth and does not model gas exchange and the effect of carbon dioxide on the oxygen dose, which results in slightly higher oxygen concentrations at the entrance to the respiratory system than would be observed in vivo. While the discussion provided above has included reference to previous literature describing oxygen delivery to adults as well as children, the present experiments were conducting using an adult airway replica only. It may be that Eqs. 1 and 2 provide reasonable estimation of oxygen delivery in pediatric and neonatal settings as well as for adults, by accounting for variation across age groups in tidal volume, inhalation time, and typical supplemental oxygen flows; however, this has not been demonstrated in the present study. Further studies incorporating pediatric and neonatal upper airway replicas [64, 65] are needed, given the importance of maintaining targeted ranges of arterial oxygen content in children [66], as in adults [67]. Additional interface types may also be relevant to the pediatric and neonatal settings.

## Conclusions

This paper has presented data describing oxygen dose in the form of concentration or flow (quantity per unit time) that was measured using a physiological realistic in vitro upper airway model. In particular, these data reveal that for low flow oxygen delivery using open patient interfaces there is great variability in the quantity, or dose, of oxygen delivered over a large range of scenarios. Among the baseline cases studied, the chief reasons for variation were 1) influence of variation in tidal volume leading to variable FiO2 and 2) variation in breathing frequency affecting volume of supplemental oxygen delivered through the breath. These data and conclusions are consistent with the understanding of expert respiratory clinicians, and they are added to the medical literature to emphasize the need for the medical community to better understand these fundamental concepts of oxygen dosing.

## Additional file

Additional file 1: **Table S1.** List of experimental cases and results. (DOCX 53 kb)

## Data Availability

All supporting data is available on request.
