# Supplementary material for: Dose variability of supplemental oxygen therapy with open patient interfaces based on in vitro measurements using a physiologically realistic upper airway model
Source: Respir Res. 2019 Jul 12;20:149. doi: 10.1186/s12931-019-1104-0 (PMC6625031; doi:10.1186/s12931-019-1104-0)
Supplement: Supplementary file 1 — Table S1. List of experimental cases and results. (DOCX 53 kb) [file 12931_2019_1104_MOESM1_ESM.docx]

**Supplemental Data**

**Table S1**. List of experimental cases and results.

| **Case #** | **Description** | **Vt (mL)** | **F (bpm)** | **i/e ratio** | **Qtotal Inhalation Flow Rate (L/min)** | **Qambient InhalationFlow Rate (L/min)** | **QO2 (L/min)** | **Linear Estimated**  **Interface FiO2%** | **Measured tracheal FtO2%** | **Interface VO2 Minute Ventilation(L/min)** | **Trachea VO2 Minute Ventilation(L/min)** | **Interface O2 Inhaled (mL/breath)** | **Trachea O2 (mL/breath)** |
| --- | --- | --- | --- | --- | --- | --- | --- | --- | --- | --- | --- | --- | --- |
| 1 | Baseline  (Hudson Straight Cannula) | 500 | 12 | 0.54 | 17.1 | 15.1 | 2 | 30.2 | 30.8 | 1.8 | 1.9 | 151 | 154 |
| 2 |  | 500 | 12 | 0.54 | 17.1 | 13.1 | 4 | 39.4 | 40.3 | 2.4 | 2.4 | 197 | 202 |
| 3 |  | 500 | 12 | 0.54 | 17.1 | 11.1 | 6 | 48.7 | 48.0 | 2.9 | 2.9 | 243 | 240 |
| 4 |  | 640 | 17 | 0.52 | 32.0 | 30.0 | 2 | 25.9 | 25.9 | 2.8 | 2.8 | 166 | 166 |
| 5 |  | 640 | 17 | 0.52 | 32.0 | 28.0 | 4 | 30.9 | 31.3 | 3.4 | 3.4 | 198 | 201 |
| 6 |  | 640 | 17 | 0.52 | 32.0 | 26.0 | 6 | 35.8 | 35.4 | 3.9 | 3.9 | 229 | 227 |
| 7 |  | 800 | 22 | 0.54 | 50.3 | 48.3 | 2 | 24.1 | 24.3 | 4.2 | 4.3 | 193 | 194 |
| 8 |  | 800 | 22 | 0.54 | 50.3 | 46.3 | 4 | 27.3 | 27.6 | 4.8 | 4.9 | 218 | 221 |
| 9 |  | 800 | 22 | 0.54 | 50.3 | 44.3 | 6 | 30.4 | 30.2 | 5.4 | 5.3 | 243 | 242 |
| 10 |  | 640 | 12 | 0.54 | 21.9 | 19.9 | 2 | 28.2 | 29.1 | 2.2 | 2.2 | 180 | 186 |
| 11 |  | 640 | 12 | 0.54 | 21.9 | 17.9 | 4 | 35.4 | 36.1 | 2.7 | 2.8 | 227 | 231 |
| 12 |  | 640 | 12 | 0.54 | 21.9 | 15.9 | 6 | 42.6 | 43.2 | 3.3 | 3.3 | 273 | 276 |
| 13 |  | 800 | 12 | 0.54 | 27.4 | 25.4 | 2 | 26.8 | 27.4 | 2.6 | 2.6 | 214 | 219 |
| 14 |  | 800 | 12 | 0.54 | 27.4 | 23.4 | 4 | 32.5 | 33.4 | 3.1 | 3.2 | 260 | 267 |
| 15 |  | 800 | 12 | 0.54 | 27.4 | 21.4 | 6 | 38.3 | 39.2 | 3.7 | 3.8 | 306 | 313 |
| 16 |  | 500 | 17 | 0.54 | 24.3 | 22.3 | 2 | 27.5 | 28.3 | 2.3 | 2.4 | 138 | 141 |
| 17 |  | 500 | 17 | 0.54 | 24.3 | 20.3 | 4 | 34.0 | 35.0 | 2.9 | 3.0 | 170 | 175 |
| 18 |  | 500 | 17 | 0.54 | 24.3 | 18.3 | 6 | 40.5 | 41.7 | 3.4 | 3.5 | 203 | 209 |
| 19 |  | 800 | 17 | 0.54 | 38.9 | 36.9 | 2 | 25.1 | 25.3 | 3.4 | 3.4 | 201 | 203 |
| 20 |  | 800 | 17 | 0.54 | 38.9 | 34.9 | 4 | 29.1 | 29.3 | 4.0 | 4.0 | 233 | 234 |
| 21 |  | 800 | 17 | 0.54 | 38.9 | 32.9 | 6 | 33.2 | 33.6 | 4.5 | 4.6 | 266 | 269 |
| 22 |  | 500 | 22 | 0.54 | 31.4 | 29.4 | 2 | 26.0 | 26.8 | 2.9 | 2.9 | 130 | 134 |
| 23 |  | 500 | 22 | 0.54 | 31.4 | 27.4 | 4 | 31.1 | 31.9 | 3.4 | 3.5 | 155 | 159 |
| 24 |  | 500 | 22 | 0.54 | 31.4 | 25.4 | 6 | 36.1 | 37.5 | 4.0 | 4.1 | 180 | 187 |
| 25 |  | 640 | 22 | 0.54 | 40.2 | 38.2 | 2 | 24.9 | 25.2 | 3.5 | 3.5 | 160 | 161 |
| 26 |  | 640 | 22 | 0.54 | 40.2 | 36.2 | 4 | 28.9 | 29.3 | 4.1 | 4.1 | 185 | 187 |
| 27 |  | 640 | 22 | 0.54 | 40.2 | 34.2 | 6 | 32.8 | 33.6 | 4.6 | 4.7 | 210 | 215 |
| 28 | Rapid Breathing  (Hudson Straight Cannula) | 250 | 30 | 1.00 | 15.0 | 13.0 | 2 | 31.5 | 31.6 | 2.4 | 2.4 | 79 | 79 |
| 29 |  | 250 | 30 | 1.00 | 15.0 | 11.0 | 4 | 42.1 | 41.9 | 3.2 | 3.1 | 105 | 105 |
| 30 |  | 250 | 30 | 1.00 | 15.0 | 9.0 | 6 | 52.6 | 50.6 | 3.9 | 3.8 | 132 | 126 |
| 31 |  | 750 | 30 | 1.00 | 45.0 | 43.0 | 2 | 24.5 | 24.6 | 5.5 | 5.5 | 184 | 184 |
| 32 |  | 750 | 30 | 1.00 | 45.0 | 41.0 | 4 | 28.0 | 27.9 | 6.3 | 6.3 | 210 | 209 |
| 33 |  | 750 | 30 | 1.00 | 45.0 | 39.0 | 6 | 31.5 | 31.4 | 7.1 | 7.1 | 237 | 235 |
| 34 | i/e = 1  (Hudson Straight Cannula) | 500 | 12 | 1.00 | 12.0 | 10.0 | 2 | 34.2 | 33.3 | 2.1 | 2.0 | 171 | 166 |
| 35 |  | 500 | 12 | 1.00 | 12.0 | 8.0 | 4 | 47.3 | 45.0 | 2.8 | 2.7 | 237 | 225 |
| 36 |  | 500 | 12 | 1.00 | 12.0 | 6.0 | 6 | 60.5 | 56.3 | 3.6 | 3.4 | 303 | 281 |
| 37 |  | 640 | 17 | 1.00 | 21.8 | 19.8 | 2 | 28.3 | 27.9 | 3.1 | 3.0 | 181 | 179 |
| 38 |  | 640 | 17 | 1.00 | 21.8 | 17.8 | 4 | 35.5 | 34.8 | 3.9 | 3.8 | 227 | 223 |
| 39 |  | 640 | 17 | 1.00 | 21.8 | 15.8 | 6 | 42.8 | 41.4 | 4.7 | 4.5 | 274 | 265 |
| 40 |  | 800 | 22 | 1.00 | 35.2 | 33.2 | 2 | 25.5 | 25.3 | 4.5 | 4.4 | 204 | 202 |
| 41 |  | 800 | 22 | 1.00 | 35.2 | 31.2 | 4 | 30.0 | 29.4 | 5.3 | 5.2 | 240 | 235 |
| 42 |  | 800 | 22 | 1.00 | 35.2 | 29.2 | 6 | 34.5 | 33.7 | 6.1 | 5.9 | 276 | 270 |
| 43 | EEP = 20%  (Hudson Straight Cannula) | 500 | 12 | 0.54 | 17.1 | 15.1 | 2 | 30.2 | 33.2 | 1.8 | 2.0 | 151 | 166 |
| 44 |  | 500 | 12 | 0.54 | 17.1 | 13.1 | 4 | 39.4 | 40.6 | 2.4 | 2.4 | 197 | 203 |
| 45 |  | 500 | 12 | 0.54 | 17.1 | 11.1 | 6 | 48.7 | 48.2 | 2.9 | 2.9 | 243 | 241 |
| 46 |  | 640 | 17 | 0.52 | 32.0 | 30.0 | 2 | 25.9 | 27.6 | 2.8 | 3.0 | 166 | 176 |
| 47 |  | 640 | 17 | 0.52 | 32.0 | 28.0 | 4 | 30.9 | 31.7 | 3.4 | 3.5 | 198 | 203 |
| 48 |  | 640 | 17 | 0.52 | 32.0 | 26.0 | 6 | 35.8 | 36.7 | 3.9 | 4.0 | 229 | 235 |
| 49 |  | 800 | 22 | 0.54 | 50.3 | 48.3 | 2 | 24.1 | 25.3 | 4.2 | 4.5 | 193 | 203 |
| 50 |  | 800 | 22 | 0.54 | 50.3 | 46.3 | 4 | 27.3 | 28.6 | 4.8 | 5.0 | 218 | 229 |
| 51 |  | 800 | 22 | 0.54 | 50.3 | 44.3 | 6 | 30.4 | 31.2 | 5.4 | 5.5 | 243 | 250 |
| 52 | EIP = 10%  (Hudson Straight Cannula) | 500 | 12 | 0.54 | 17.1 | 15.1 | 2 | 30.2 | 30.9 | 1.8 | 1.9 | 151 | 154 |
| 53 |  | 500 | 12 | 0.54 | 17.1 | 13.1 | 4 | 39.4 | 39.1 | 2.4 | 2.3 | 197 | 196 |
| 54 |  | 500 | 12 | 0.54 | 17.1 | 11.1 | 6 | 48.7 | 46.9 | 2.9 | 2.8 | 243 | 235 |
| 55 |  | 640 | 17 | 0.52 | 32.0 | 30.0 | 2 | 25.9 | 26.1 | 2.8 | 2.8 | 166 | 167 |
| 56 |  | 640 | 17 | 0.52 | 32.0 | 28.0 | 4 | 30.9 | 30.5 | 3.4 | 3.3 | 198 | 195 |
| 57 |  | 640 | 17 | 0.52 | 32.0 | 26.0 | 6 | 35.8 | 35.4 | 3.9 | 3.8 | 229 | 226 |
| 58 |  | 800 | 22 | 0.54 | 50.3 | 48.3 | 2 | 24.1 | 24.4 | 4.2 | 4.3 | 193 | 195 |
| 59 |  | 800 | 22 | 0.54 | 50.3 | 46.3 | 4 | 27.3 | 27.3 | 4.8 | 4.8 | 218 | 218 |
| 60 |  | 800 | 22 | 0.54 | 50.3 | 44.3 | 6 | 30.4 | 30.3 | 5.4 | 5.3 | 243 | 242 |
| 61 | Hudson Flared Cannula | 500 | 12 | 0.54 | 17.1 | 15.1 | 2 | 30.2 | 30.3 | 1.8 | 1.8 | 151 | 152 |
| 62 |  | 500 | 12 | 0.54 | 17.1 | 13.1 | 4 | 39.4 | 39.2 | 2.4 | 2.4 | 197 | 196 |
| 63 |  | 500 | 12 | 0.54 | 17.1 | 11.1 | 6 | 48.7 | 47.5 | 2.9 | 2.8 | 243 | 237 |
| 64 |  | 640 | 17 | 0.52 | 32.0 | 30.0 | 2 | 25.9 | 25.9 | 2.8 | 2.8 | 166 | 166 |
| 65 |  | 640 | 17 | 0.52 | 32.0 | 28.0 | 4 | 30.9 | 31.0 | 3.4 | 3.4 | 198 | 198 |
| 66 |  | 640 | 17 | 0.52 | 32.0 | 26.0 | 6 | 35.8 | 35.6 | 3.9 | 3.9 | 229 | 228 |
| 67 |  | 800 | 22 | 0.54 | 50.3 | 48.3 | 2 | 24.1 | 24.4 | 4.2 | 4.3 | 193 | 195 |
| 68 |  | 800 | 22 | 0.54 | 50.3 | 46.3 | 4 | 27.3 | 27.4 | 4.8 | 4.8 | 218 | 219 |
| 69 |  | 800 | 22 | 0.54 | 50.3 | 44.3 | 6 | 30.4 | 30.7 | 5.4 | 5.4 | 243 | 246 |
| 70 | Salter Straight Cannula | 500 | 12 | 0.54 | 17.1 | 15.1 | 2 | 30.2 | 31.3 | 1.8 | 1.9 | 151 | 157 |
| 71 |  | 500 | 12 | 0.54 | 17.1 | 13.1 | 4 | 39.4 | 39.5 | 2.4 | 2.4 | 197 | 198 |
| 72 |  | 500 | 12 | 0.54 | 17.1 | 11.1 | 6 | 48.7 | 49.5 | 2.9 | 3.0 | 243 | 247 |
| 73 |  | 640 | 17 | 0.52 | 32.0 | 30.0 | 2 | 25.9 | 26.5 | 2.8 | 2.9 | 166 | 170 |
| 74 |  | 640 | 17 | 0.52 | 32.0 | 28.0 | 4 | 30.9 | 31.3 | 3.4 | 3.4 | 198 | 200 |
| 75 |  | 640 | 17 | 0.52 | 32.0 | 26.0 | 6 | 35.8 | 37.3 | 3.9 | 4.1 | 229 | 239 |
| 76 |  | 800 | 22 | 0.54 | 50.3 | 48.3 | 2 | 24.1 | 24.4 | 4.2 | 4.3 | 193 | 195 |
| 77 |  | 800 | 22 | 0.54 | 50.3 | 46.3 | 4 | 27.3 | 27.8 | 4.8 | 4.9 | 218 | 222 |
| 78 |  | 800 | 22 | 0.54 | 50.3 | 44.3 | 6 | 30.4 | 31.2 | 5.4 | 5.5 | 243 | 249 |
| 79 | Simple Oxygen Mask | 500 | 12 | 0.54 | 17.1 | 15.1 | 2 | 30.2 | 33.3 | 1.8 | 2.0 | 151 | 166 |
| 80 |  | 500 | 12 | 0.54 | 17.1 | 13.1 | 4 | 39.4 | 42.0 | 2.4 | 2.5 | 197 | 210 |
| 81 |  | 500 | 12 | 0.54 | 17.1 | 11.1 | 6 | 48.7 | 50.5 | 2.9 | 3.0 | 243 | 252 |
| 82 |  | 640 | 17 | 0.52 | 32.0 | 30.0 | 2 | 25.9 | 28.2 | 2.8 | 3.1 | 166 | 180 |
| 83 |  | 640 | 17 | 0.52 | 32.0 | 28.0 | 4 | 30.9 | 33.5 | 3.4 | 3.6 | 198 | 215 |
| 84 |  | 640 | 17 | 0.52 | 32.0 | 26.0 | 6 | 35.8 | 38.2 | 3.9 | 4.2 | 229 | 244 |
| 85 |  | 800 | 22 | 0.54 | 50.3 | 48.3 | 2 | 24.1 | 25.3 | 4.2 | 4.5 | 193 | 203 |
| 86 |  | 800 | 22 | 0.54 | 50.3 | 46.3 | 4 | 27.3 | 28.9 | 4.8 | 5.1 | 218 | 231 |
| 87 |  | 800 | 22 | 0.54 | 50.3 | 44.3 | 6 | 30.4 | 32.0 | 5.4 | 5.6 | 243 | 256 |
| 88 | Southmedic Oxymask | 500 | 12 | 0.54 | 17.1 | 15.1 | 2 | 30.2 | 33.0 | 1.8 | 2.0 | 151 | 165 |
| 89 |  | 500 | 12 | 0.54 | 17.1 | 13.1 | 4 | 39.4 | 41.0 | 2.4 | 2.5 | 197 | 205 |
| 90 |  | 500 | 12 | 0.54 | 17.1 | 11.1 | 6 | 48.7 | 45.8 | 2.9 | 2.7 | 243 | 229 |
| 91 |  | 640 | 17 | 0.52 | 32.0 | 30.0 | 2 | 25.9 | 26.8 | 2.8 | 2.9 | 166 | 171 |
| 92 |  | 640 | 17 | 0.52 | 32.0 | 28.0 | 4 | 30.9 | 32.8 | 3.4 | 3.6 | 198 | 210 |
| 93 |  | 640 | 17 | 0.52 | 32.0 | 26.0 | 6 | 35.8 | 37.9 | 3.9 | 4.1 | 229 | 242 |
| 94 |  | 800 | 22 | 0.54 | 50.3 | 48.3 | 2 | 24.1 | 24.6 | 4.2 | 4.3 | 193 | 197 |
| 95 |  | 800 | 22 | 0.54 | 50.3 | 46.3 | 4 | 27.3 | 28.2 | 4.8 | 5.0 | 218 | 225 |
| 96 |  | 800 | 22 | 0.54 | 50.3 | 44.3 | 6 | 30.4 | 31.7 | 5.4 | 5.6 | 243 | 253 |
| 97 | Southmedic Oxyarm | 500 | 12 | 0.54 | 17.1 | 15.1 | 2 | 30.2 | 29.6 | 1.8 | 1.8 | 151 | 148 |
| 98 |  | 500 | 12 | 0.54 | 17.1 | 13.1 | 4 | 39.4 | 38.2 | 2.4 | 2.3 | 197 | 191 |
| 99 |  | 500 | 12 | 0.54 | 17.1 | 11.1 | 6 | 48.7 | 42.9 | 2.9 | 2.6 | 243 | 214 |
| 100 |  | 640 | 17 | 0.52 | 32.0 | 30.0 | 2 | 25.9 | 25.9 | 2.8 | 2.8 | 166 | 166 |
| 101 |  | 640 | 17 | 0.52 | 32.0 | 28.0 | 4 | 30.9 | 30.6 | 3.4 | 3.3 | 198 | 196 |
| 102 |  | 640 | 17 | 0.52 | 32.0 | 26.0 | 6 | 35.8 | 33.7 | 3.9 | 3.7 | 229 | 215 |
| 103 |  | 800 | 22 | 0.54 | 50.3 | 48.3 | 2 | 24.1 | 24.2 | 4.2 | 4.3 | 193 | 194 |
| 104 |  | 800 | 22 | 0.54 | 50.3 | 46.3 | 4 | 27.3 | 26.9 | 4.8 | 4.7 | 218 | 215 |
| 105 |  | 800 | 22 | 0.54 | 50.3 | 44.3 | 6 | 30.4 | 30.4 | 5.4 | 5.3 | 243 | 243 |
